# Case report: Right vertebral and carotid artery anomalies with an aberrant right subclavian artery in two patients

**DOI:** 10.3389/fneur.2023.1282127

**Published:** 2023-12-13

**Authors:** Jiang Chen, LunXin Liu, Xiaobo Kou, Chaohua Wang

**Affiliations:** ^1^Department of Neurosurgery, West China Hospital of Sichuan University, Chengdu, China; ^2^Department of Neurosurgery, Dazhu Hospital, Dazhou, China; ^3^Department of Neurosurgery, The First People’s Hospital in Shuangliu District/West China Airport Hospital, Sichuan University, Chengdu, China

**Keywords:** right subclavian artery, anomalous origin, right vertebral artery, aberrance, aortic arch

## Abstract

Abnormal origins of the vertebral artery with supra-aortic vessel variants are exceedingly uncommon. Herein, we present two cases of the vertebral artery originating from the right common carotid artery associated with the right subclavian artery arising separately as the initial branch of the aortic arch, followed by the right common carotid artery. We reviewed the embryology of the anomalous origins of the vertebral and subclavian arteries. These variants can significantly affect surgical planning and cause severe clinical symptoms.

## Introduction

Abnormal origins of the right vertebral artery (RVA) are uncommon, occurring at a rate of 0.18% in the general population (1). Right subclavian artery (RSCA) variants are primarily observed in vascular ring anomalies of the aortic arch, with incidence rates ranging from 0.5% to 2% (2). Therefore, RVA anomalies with an aberrant RSCA are extremely rare. To the best of our knowledge, an abnormal origin of the RVA associated with the RSCA arising as the first branch of the aortic arch has not been described in the literature (1, 3). We present two cases of the RVA originating from the right common carotid artery (RCCA), associated with the RSCA arising separately as the initial branch of the aortic arch, followed by the RCCA.

## Case reports

### Case 1

**Figure 1:**
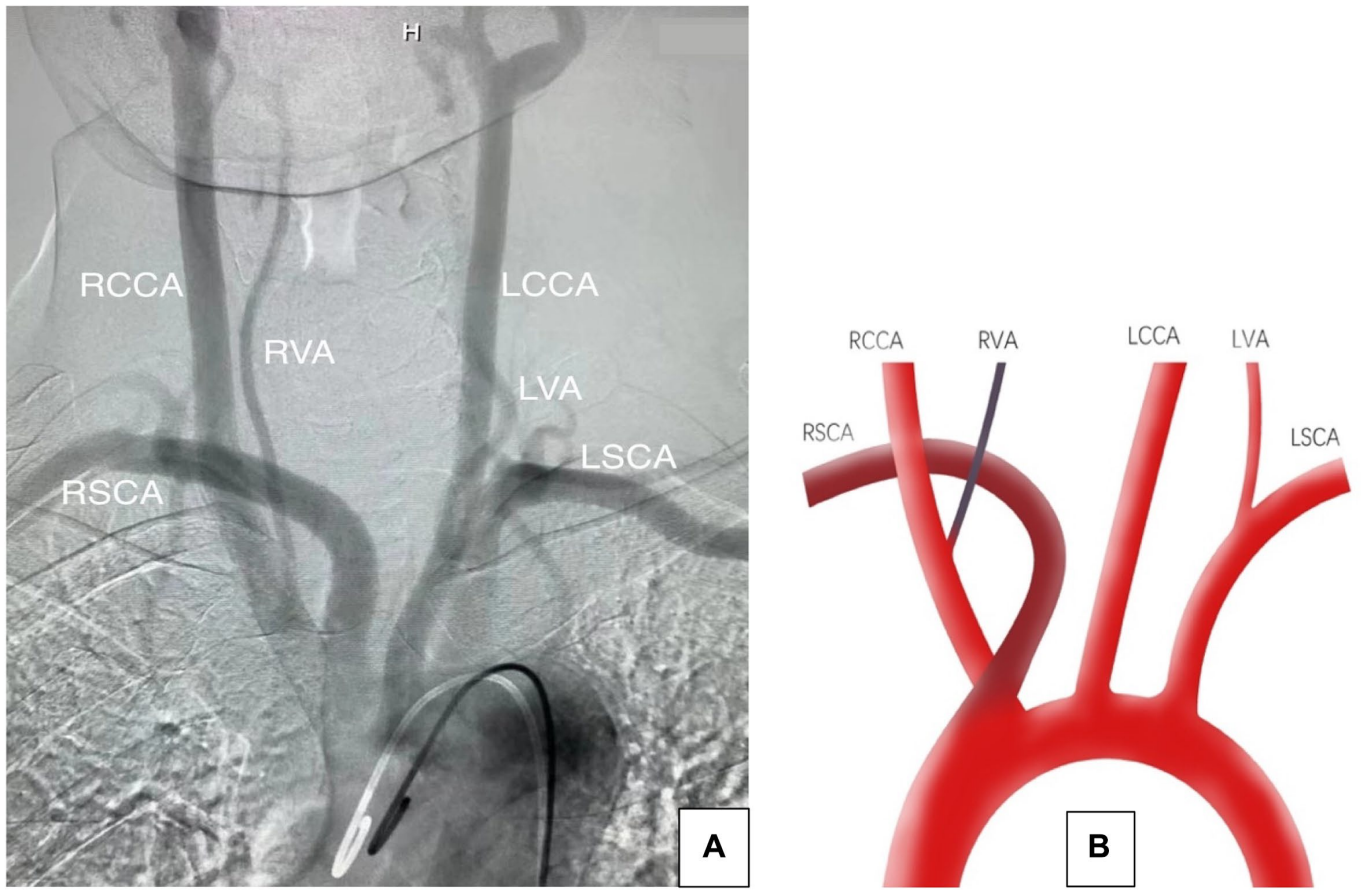
**(A)** Aortic arch angiogram showing the aberrant origin of the right subclavian artery and the right vertebral artery. **(B)** Illustration showing that the RVA originated from the RCCA, both the RCCA and the RSCA originated directly from the aortic arch, and the RSCA was the first branch, followed by the RCCA. LCCA, left common carotid artery; LSCA, left subclavian artery; RVA, right vertebral artery; RCCA, right common carotid artery; RSCA, right subclavian artery; LVA, left vertebral artery.

A 65 years-old woman presented with a history of acute severe headaches the previous year after an emotional trauma. She has no significant family or past medical history. Brain CT revealed a diffuse subarachnoid hemorrhage and a right posterior communicating aneurysm (PComA) was seen on CT angiogram. This patient was successfully treated with microsurgical clipping. An initial cerebral angiogram was performed for follow-up. Digital subtraction angiography (DSA) imaging demonstrated that the RVA arose from the RCCA and that the RSCA was the first branch arising from the aortic arch, followed by the RCCA (see Figure 1).

### Case 2

**Figure 2:**
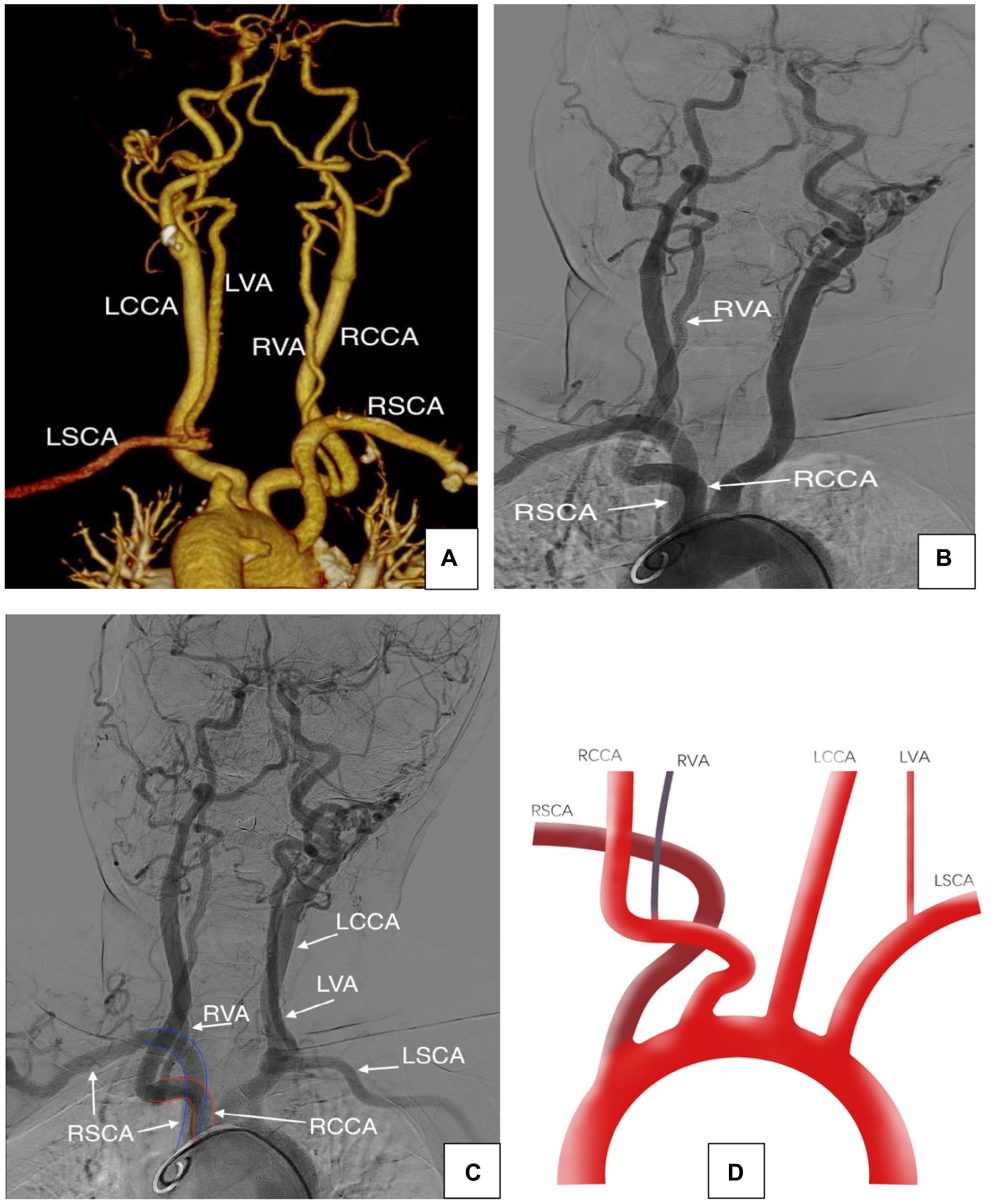
**(A–C)** Arch CTA and DSA showing the aberrant origin of the right subclavian artery and the right vertebral artery. **(D)** Schematic representation of the case showing that the RVA originated from RCCA and the RSCA was the first branch to arise from the aortic arch, followed by the RCCA.

A 60 years-old man with hypertension presented with a history of severe acute-onset headaches. Computed tomography angiography (CTA) of the head identified a left PComA aneurysm and a subarachnoid hemorrhage. In addition, arch CTA demonstrated that the RVA originated from the RCCA, part of the left subclavian artery (LSCA) was missing, the brachiocephalic trunk was absent, the RCCA and the RSCA had separate origins from the aortic arch, and the RSCA was the first branch to emerge, followed by the RCCA. DSA was performed and demonstrated that the LSCA had diverted blood from the RVA. A coiling procedure successfully treated the left PComA aneurysm and the patient’s neurological condition was excellent at discharge. No intervention was performed for the LSCA, and the patient was managed with an antiplatelet regimen when necessary (see Figure 2).

## Discussion

In a typical aortic arch anatomy, the vertebral arteries are conventionally identified as the primary branches of the subclavian arteries. The most common variation of the vertebral arteries is in the left vertebral artery, with an incidence rate of 2.4%–5.8% (3, 4). In contrast, variations in the origin of the RVA are less common, with an incidence rate of 0.18% (1). If the RVA arises from the RCCA, its association with RSCA abnormalities also increases (5–8). Although the embryology of abnormal RVA and RSCA is complex, understanding embryonic development and branching patterns can help explain these abnormalities.

**Figure 3:**
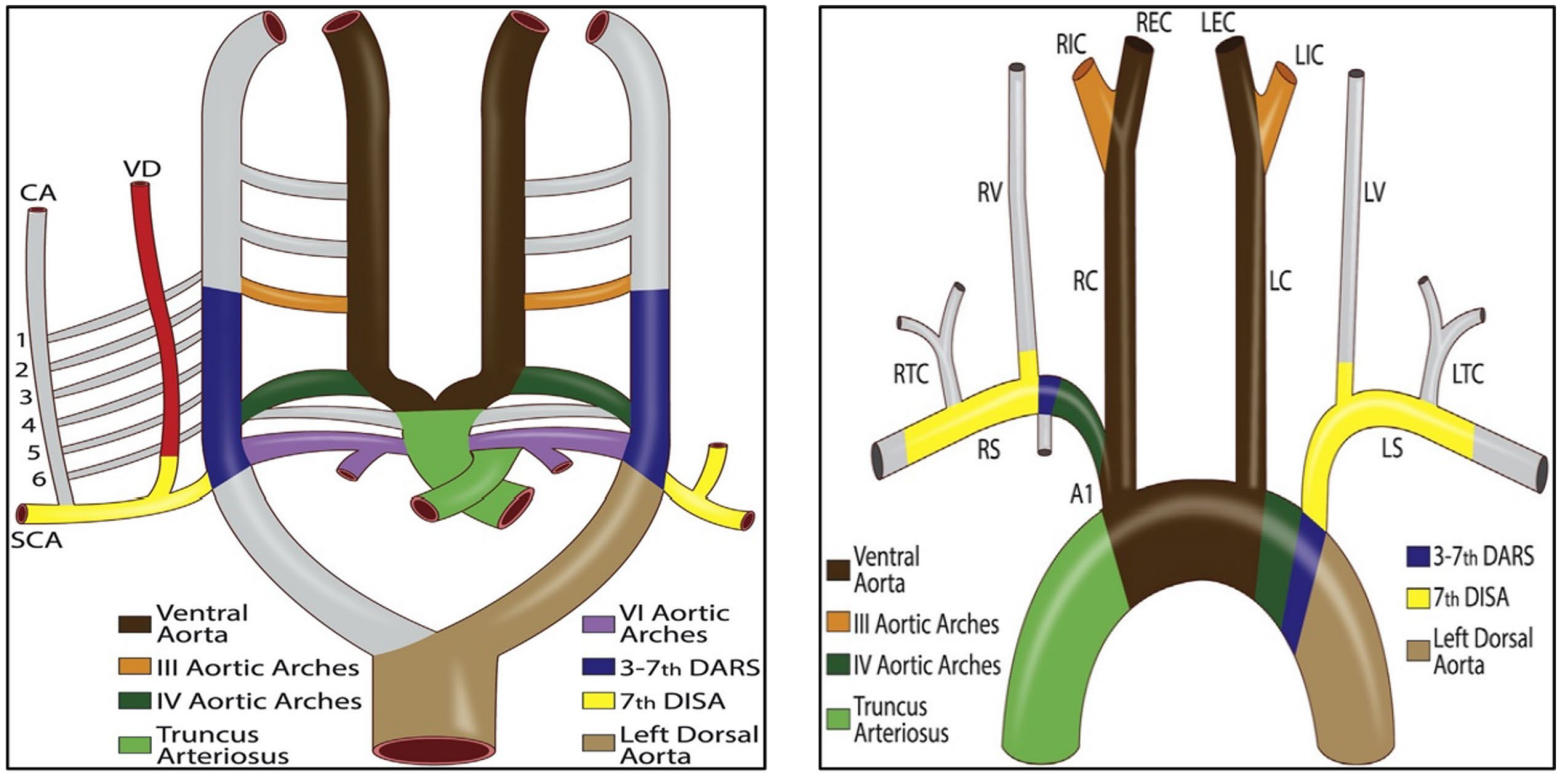
Schematic diagram of the primitive ventral and dorsal aorta (1). DARS, dorsal aortic root segments; VD, right vertebral artery; CA, ascending cervical artery; DISA, dorsal intersegmental arteries.

During the early stages of embryonic development, the right ventral aorta forms the right common and brachiocephalic arteries. At the 7 mm stage, seven cervical intersegmental arteries (CIA) emerge on the aortic arch (9–11). At the 10–12 mm stage, the first to sixth CIAs anastomose longitudinally to form the VA (12). At the 14–17 mm stage, the horizontal segments of the first six CIAs degenerate and disappear, and the seventh CIA combined with the fourth aortic arch form the subclavian artery (5). Thus, the VA commonly arises from the subclavian artery, which is the largest and most proximal branch (Figure 3).

**Figure 4:**
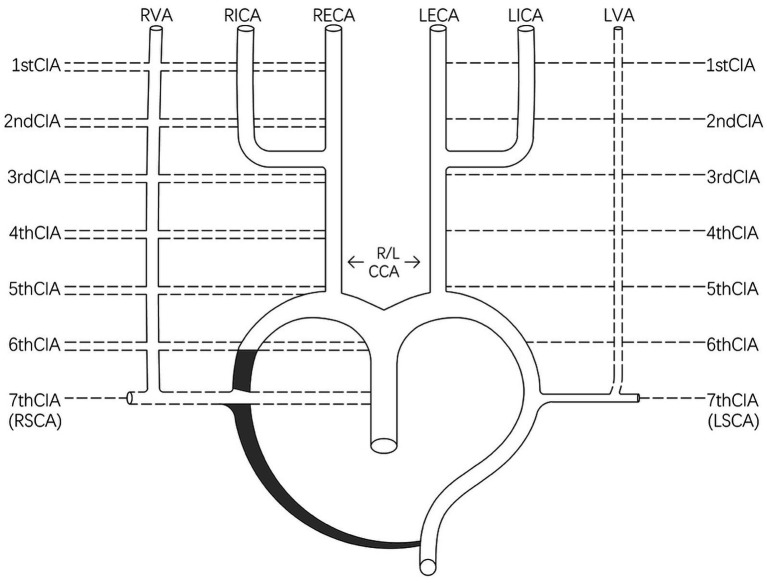
Schematic of the various anomalous origins of the VA. CIA, cervical intersegmental artery; R/LCCA, right/left common carotid artery; RICA, right internal carotid artery; LICA, left internal carotid artery; RECA, right external carotid artery; LECA, left external carotid artery.

Nevertheless, incomplete involution of one of the first six CIAs leads to various anomalous origins of the VA (13). If the horizontal segments of the first or second CIA persist, an aberrant VA origin may develop in the external or internal carotid artery. If the third, fourth, or fifth CIA persist, the VA may originate from the RCCA. If the sixth CIA persists, an abnormal VA origin may arise from the aortic arch (Figure 4).

In addition, at the 40 mm stage of embryonic development, the remaining vessel of the right third aortic arch combines with the right half of the fourth ventral aorta to form the brachiocephalic trunk (BT). The possible cause of missing the BT is that the seventh CIA originates directly from the ascending aorta or that the right aortic arch partially degenerates, resulting in separate emergence of the RCCA and RSCA. The RSCA arises first, followed by the RCCA (Figure 4).

Although RVA and RSCA variations are only anatomical, and most are asymptomatic, it is essential to recognize these rare anomalies before surgery. When the RVA originates anomalously from the RCCA and its branches, injury to, or intraoperative blockage of, the corresponding carotid artery during surgery may cause fatal brain ischemia. When the RSCA originates anomalously from the aortic arch, surgical procedures and endovascular interventions involving the arch may pose significant challenges. Additionally, the angles of the opening of the variant RVA and RSCA are always tricky, and super selection for endovascular treatment is often difficult. Therefore, detailed information on these anatomical variations is of broad concern to clinicians.

## Conclusion

The RVA from an RCCA associated with an RSCA arising as the first branch, followed by the RCCA, is extremely rare. The embryology of abnormal RVA and RSCA is complex. Physicians should identify these abnormalities before surgery.

## Data availability statement

The original contributions presented in the study are included in the article/supplementary material, further inquiries can be directed to the corresponding author.

## Ethics statement

Ethical review and approval was not required for the study on human participants in accordance with the local legislation and institutional requirements. Written informed consent from the patients/participants or patients/participants’ legal guardian/next of kin was not required to participate in this study in accordance with the national legislation and the institutional requirements. Written informed consent was obtained from the individual(s) for the publication of any potentially identifiable images or data included in this article.

## Author contributions

JC: Writing – original draft, Writing – review & editing. LL: Formal analysis, Methodology, Writing – review & editing. XK: Data curation, Writing – review & editing. CW: Data curation, Formal analysis, Methodology, Supervision, Writing – review & editing.
